# Assessment of Ileal Crohn’s Disease Activity by Gastrointestinal Ultrasound and MR Enterography: A Pilot Study

**DOI:** 10.3390/life13081754

**Published:** 2023-08-16

**Authors:** Răzvan-Cristian Statie, Sevastița Iordache, Lucian Mihai Florescu, Ioana-Andreea Gheonea, Victor-Mihai Sacerdoțianu, Bogdan Silviu Ungureanu, Ion Rogoveanu, Dan-Ionuț Gheonea, Tudorel Ciurea, Dan Nicolae Florescu

**Affiliations:** 1 Doctoral School, University of Medicine and Pharmacy of Craiova, 200349 Craiova, Romania; statierazvan@gmail.com; 2 Department of Gastroenterology, University of Medicine and Pharmacy of Craiova, 200349 Craiova, Romania; 3 Department of Radiology and Medical Imaging, University of Medicine and Pharmacy of Craiova, 200349 Craiova, Romania

**Keywords:** Crohn’s disease, intestinal ultrasonography, contrast-enhanced ultrasound, MR enterography

## Abstract

Introduction: In some cases, there may be a discrepancy between the symptomatology alleged by Crohn’s disease (CD) patients and the results of laboratory tests or imaging investigations. Ileocolonoscopy with biopsy is the primary investigation for diagnosing and monitoring CD patients. Cross-sectional imaging techniques such as CT or MR enterography (MRE) and intestinal ultrasonography (IUS) have been proposed as complementary methods to colonoscopy for a complete evaluation of this category of patients. This study aims to identify the role of IUS, contrast-enhanced ultrasound (CEUS) and MRE in evaluating ileal CD activity, using clinical severity scores (Crohn’s disease activity index—CDAI, Harvey–Bradshaw index—HBI) and faecal calprotectin or C-reactive protein (CRP) levels as reference methods. Materials and Methods: A total of 44 adult patients with ileal CD confirmed using an ileocolonoscopy with biopsy and histopathological examination were assessed by IUS, CEUS and MRE. The evaluation of the disease activity based on the results obtained from the cross-sectional imaging tests was carried out by using some severity scores available in the literature. The sensitivity and specificity of IUS + CEUS and MRE for differentiating active from inactive forms of CD were determined using CDAI, HBI, faecal calprotectin and CRP as reference methods. The accuracy of the results was assessed by the receiver operating characteristics method. The Pearson correlation coefficient was used to determine the types of correlation. A *p*-value less than 0.05 suggested a statistically significant relationship. Results: Compared to CDAI, the best correlation was identified for Limberg score (r = 0.667, 95% confidence interval (CI) [0.46, 0.8], *p* < 0.001), followed by MaRIAs score (r = 0.614, 95% CI [0.39, 0.77], *p* < 0.001). A sensitivity of 93.33% and a specificity of 71.43% (AUC = 0.98) were demonstrated in the case of Limberg score for differentiating patients with active disease from those in remission and for MaRIAs score a sensitivity of 100.00% and a specificity of 57.14% (AUC = 0.97). Regarding HBI, the best correlation was observed for MaRIAs score (r = 0.594, 95% CI [0.36, 0.76], *p* < 0.001). Also, faecal calprotectin showed the best correlation with MaRIAs score (r = 0.697, 95% CI [0.46, 0.84], *p* < 0.001), but in the case of CRP, there was only a weak correlation for all evaluated scores. Conclusions: Although magnetic resonance imaging does not appear to be superior to ultrasonography in terms of accuracy for differentiating active forms of CD from those in remission, the results of our study suggest that MRE associates a better correlation with clinical severity scores and faecal calprotectin levels compared to ultrasonography. More studies are needed to validate these results.

## 1. Introduction

Crohn’s disease (CD) is one of the primary forms of inflammatory bowel disease (IBD). The characteristic inflammatory process of CD is transmural, can affect all layers of the bowel wall and can spread to adjacent tissues. Regarding location, CD may interest any segment of the digestive tract. A predilection for the terminal ileum and proximal colon is noted [1]. Approximately 40% of CD patients present with ileocolonic localisation, 30% have a localisation at the colon level and 25% at the small bowel level [2]. Cases with extensive lesions in the small bowel seem to be associated with a less favourable outcome, with reduced response to drug therapy and requiring surgery and higher healthcare costs [1,3]. For this reason, properly managing these patients requires a more complex evaluation of the entire digestive tract with the correct establishment of the extent of the disease.

Ileocolonoscopy with biopsy is the primary investigation for diagnosing and monitoring CD patients [4]. The limitations of this method refer to the fact that it does not always allow endoscopic exploration of the terminal ileum, and the examination is limited to the appearance of the mucosa without providing information on the deeper layers of the bowel wall. Moreover, it is not an investigation accepted by patients to be repeated frequently [5].

Imaging evaluation of the small bowel is required in all patients in whom the diagnosis of CD is suspected [4]. Thus, cross-sectional imaging techniques such as CT enterography (CTE) or MR enterography (MRE) and intestinal ultrasonography (IUS) have been proposed as complementary methods to colonoscopy for a complete evaluation of this category of patients [6]. The computed tomography (CT) scan of the small bowel allows for obtaining high-resolution images in a shorter time compared to MRE, reducing the discomfort created for certain patients (e.g., those with claustrophobia); this method uses ionising radiation, which is why it cannot be performed repeatedly, especially in young patients [6]. MRE shows superior sensitivity and specificity to IUS in evaluating CD extension in the small bowel [4,7]. Therefore, MRE is considered the best non-ionising imaging investigation for evaluating CD patients [6,8,9]. But this method is not widely available, involves relatively high costs and sometimes patients hardly tolerate the examination [10]. Furthermore, a series of meta-analyses revealed no significant differences in diagnostic accuracy in CD between MRE, CTE or IUS [4,11,12,13,14,15]. Based on these results, IUS could represent an effective alternative to MRE for evaluating CD patients. Ultrasonography is widely available, relatively inexpensive and does not use ionising radiation, and is, thus, of great interest, especially for young patients who require frequent monitoring. But it is an operator-dependent method, which can associate difficulties in obtaining quality images in the case of obese patients and the experience in evaluating the digestive tract is limited. However, despite these impediments, recent years have seen an increase in the popularity of ultrasonography for evaluating patients with IBD.

Over time, several methods have been proposed for evaluating CD activity. Among these, we mention clinical severity scores, such as Crohn’s disease activity index (CDAI) [16] and the Harvey–Bradshaw index (HBI) [17], or laboratory tests, such as faecal calprotectin or C-reactive protein (CRP) levels [18,19,20]. The assessment of inflammation with the help of cross-sectional imaging techniques can be achieved by measuring the thickness of the bowel wall [10,11,21,22] and evaluating the mural blood flow using the colour Doppler during US examination [10,23,24,25] or by the bowel wall enhancement of the contrast agent in the case of CT or magnetic resonance imaging (MRI) [26,27]. Another parameter with an essential role in the imaging assessment of inflammation severity is the appearance of mesenteric fat [28]. Using contrast agents during US examination (contrast-enhanced ultrasound—CEUS) allows the evaluation of the microcirculation at the level of the bowel wall [10,29]. According to the EFSUMB guidelines [30], CEUS can be used to assess the activity of IBD and also allows the differentiation of inflammatory stenoses from fibrous ones in the case of CD; it can also be used to monitor treatment response in CD. Several scores have been proposed to evaluate CD activity using CEUS [10,31,32] or MRE [33], which seem to show excellent correlations with disease severity assessed by colonoscopy [34]. However, in some instances, there is a contradiction between the symptomatology alleged by the patient and the results of laboratory tests or imaging investigations.

Thus, this study aims to identify the role of IUS, CEUS and MRE in evaluating ileal CD activity, using clinical severity scores (CDAI, HBI) and faecal calprotectin or CRP levels as reference methods.

## 2. Materials and Methods

### 2.1. Study Population

This prospective study was conducted over a period of 27 months. From November 2020 to January 2023, a total of 44 adult patients with ileal CD confirmed by ileocolonoscopy with biopsy and histopathological examination were included in the study.

Inclusion criteria for patients: (1) over 18 years of age; (2) known ileal CD confirmed by histopathological examination; and (3) signed written informed consent.

Patients with general contraindications for MRE (e.g., claustrophobia, presence of metal implants); those with contraindications for contrast agents used for US or MRE; patients in whom difficulties were encountered in obtaining high-quality images during the US examination; patients who associated other diseases that could have influenced the results of laboratory tests; pregnant women; and patients who refused to participate in the study were excluded.

The interval between the two cross-sectional imaging examinations, US and MRE, was a maximum of 7 days.

The study was performed according to the principles of the Declaration of Helsinki. The local ethics committee approved the study protocol, and written informed consent was obtained for all patients before inclusion in the study.

### 2.2. Clinical Activity Scores and Laboratory Tests

When patients presented for ultrasonographic examination, they were assessed by CDAI (<150—remission) and HBI (<5—remission) activity scores. Blood and stool samples were also collected from the patients to determine hematocrit levels (necessary for CDAI calculation), CRP and faecal calprotectin. We used threshold values of 10 mg/L for CRP, respectively, and 250 µg/g for faecal calprotectin to differentiate inactive forms of the disease from active ones [35,36].

### 2.3. Ultrasound

**Figure A1:**
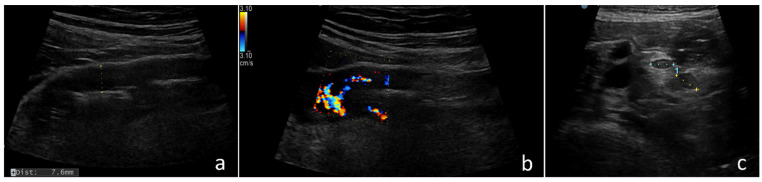
(**a**) Thickened wall (7.6 mm) with normal bowel wall stratification loss. (**b**) Colour Doppler imaging of affected segment shows abundant mural blood flow—Limberg score = grade 3. (**c**) The presence of enlarged lymph nodes.

The examination was performed by a gastroenterologist with about 15 years of experience in IUS using a Hitachi Arietta V70 ultrasonography system (Hitachi Ltd., Tokyo, Japan) and the L34 linear transducer with a frequency of 7.5 MHz. Patients were instructed to fast for at least 6 h before the procedure. The ultrasound examination was performed at the level of the terminal ileum. The assessed parameters included: maximum bowel wall thickness (BWT), parietal stratification, mural blood flow on colour Doppler imaging (CDI), mesenteric fatty proliferation and the presence of lymph nodes (Appendix A Figure A1). Considering BWT and relying on a classification proposed by Rigazio et al. [37], CD could be divided as follows: inactive, BWT < 4 mm; mild, BWT 4 mm-6 mm; moderate, BWT 6.1 mm–8 mm; and severe, BWT ≥ 8.1 mm. To assess the mural blood flow at the CDI, we used the Limberg score [23].

**Figure A2:**
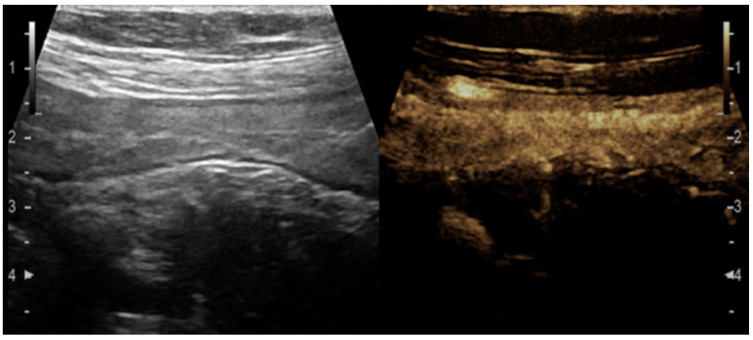
Dual-screen ultrasound representation of an abnormal segment of bowel, in grey-scale imaging (B-mode) (**left**) and contrast-enhanced ultrasound (CEUS) of the matching segment (**right**). CEUS shows transmural enhancement.

Next, an intravenous bolus injection of 4.8 mL of a second-generation contrast agent (SonoVue, Bracco S.p.A., Milan, Italy) was administered, followed by an infusion of 5 mL sodium chloride 0.9%. CEUS examinations (T0-T180s) were evaluated in real-time (Appendix A Figure A2) and then recorded on an external storage drive for quantitative analysis of the obtained images.

**Figure 1:**
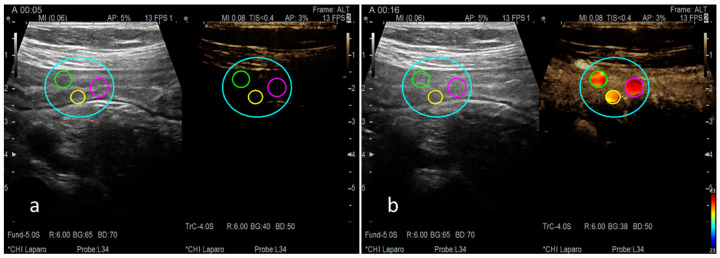
(**a**) Quantitative analysis of vascular pattern by manually choosing specific regions of interest (the three small circles) in the terminal ileum using dedicated software, Vuebox^®^ (https://www.bracco.com/en-se/product/vuebox), resulting in TIC parameters. (**b**) An example of one of the automatically calculated parameters, peak enhancement.

**Figure 2:**
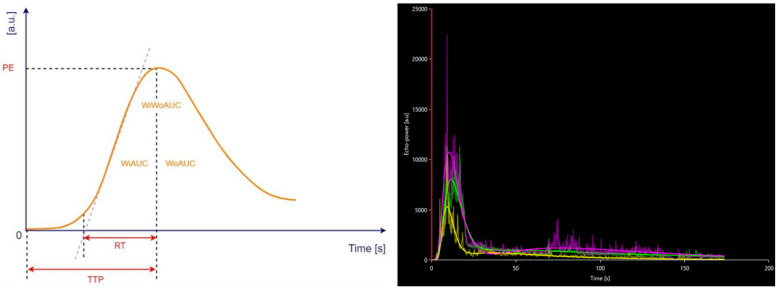
Time-intensity curve with generated parameters: peak enhancement (PE); wash-in area under the curve (WiAUC); rise time (RT); time to peak (TTP); wash-out area under the curve (WoAUC); wash-in and wash-out area under the curve (WiWoAUC). a.u., arbitrary units; s, seconds. Adapted from VueBox^®^ Quantification Toolbox User Manual, Copyright© 2019 Bracco Suisse SA.

**Figure 3:**
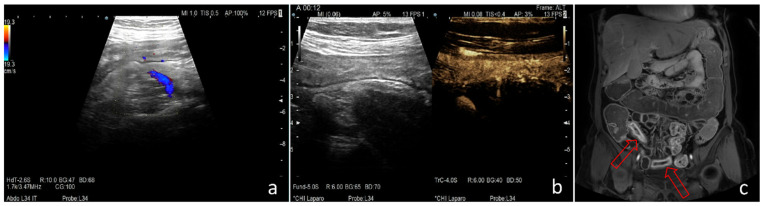
Comparison between the different scores for evaluating disease activity using IUS + CEUS or MRE in the case of a patient with a mild form of Crohn’s disease (CDAI = 152; HBI = 5; fecal calprotectin = 390 μg/g; CRP = 1.42 mg/L). (**a**) Colour Doppler imaging shows intermittent increases in vascularity—Limberg score = 2. (**b**) CEUS highlights transmural enhancement (Peak Enhancement = 38.86 dB, Wash-in Rate = 1750.75 [a.u], after the quantitative analysis of vascular pattern using VueBox^®^)—Ripollés score = 72.31, Medellin-Kowalewski score = 38.86. (**c**) MRE—T1 postcontrast phase (venous phase), coronal plane: the arrows indicate an increased wall thickness affecting the terminal ileum continuously, mainly with a mucosal contrast enhancement pattern in this phase and engorged vasa recta in the affected region—MaRIAs score = 3. In this case, we notice a discrepancy between the results of the imaging tests that suggest a severe form of CD and the results of the clinical activity scores and laboratory tests.

The quantitative analysis of the vascular pattern at the level of the wall of the terminal ileum was performed using a dedicated software called VueBox^®^ (Bracco Suisse SA, Plan-les-Ouates, Switzerland). Images recorded during CEUS were converted to DICOM format. Afterward, they were analysed through the mentioned dedicated software. Three regions of interest (ROI) were selected at the level of the terminal ileum wall—Figure 1. The time-intensity curve (TIC) was analysed with the automatic generation of the following parameters—Figure 2, as follows:

- Peak Enhancement (PE)—Figure 3b;
- Wash-in Area Under the Curve (WiAUC);
- Rise Time (RT);
- Mean Transit Time Local (mTTl);
- Time To Peak (TTP);
- Wash-in Rate (WiR);
- Wash-in Perfusion Index (WiPI);
- Wash-out AUC (WoAUC);
- Wash-in and Wash-out AUC (WiWoAUC);
- Fall Time (FT);
- Wash-out Rate (WoR);
- Quality Of Fit between the echo-power signal and f(t) (QOF).

The quality of this process was assessed based on the value of the QOF parameter, which is considered appropriate if it exceeded the 50% threshold. The results of the generated parameters were expressed in arbitrary units [a.u] and seconds [s]. To convert [a.u.] to decibels [dB], we used the following formula suggested by the developer: value_dB = 10 × log10 (value_au/1).

The evaluation of the disease activity based on the results obtained from the ultrasonographic examination was carried out by using some severity scores available in the literature. These scores used colonoscopy as a reference method, resulting in good correlations. The first score used was proposed by Medellin-Kowalewski et al. [10] and evaluates the PE parameter obtained following CEUS quantification. Thus, a value of PE < 18.2 dB seems to associate with an inactive form of the disease, values between 18.2–22.8 dB seem to characterise mild to moderate forms, and a value above 22.8 dB suggests moderate to severe forms. Ripollés et al. [31] developed an ultrasonographic score based on grey-scale ultrasound and CEUS parameters. It uses the BWT, the colour Doppler grade based on the Limberg score and the WiR parameter (expressed in [a.u]) obtained after CEUS quantification, according to the following formula (parietal thickness × 0.957) + (colour Doppler grade × 0.859) + (wash-in × 0.036). A cut-off value of 8.38 was established to distinguish inactive from active forms of the disease. They also proposed a simplified variant—simple CEUS score: thickness (mm) + colour Doppler grade + (wash-in × 0.036) (with the same cut-off value of 8.38) or a score based only on the BWT and colour Doppler grade—simple US score: parietal thickness (mm) + colour Doppler grade, using a cut-off value of 5.5. The PE and WiR values we introduced in the formulas mentioned above were represented by the average of these parameters obtained at the level of each ROI.

### 2.4. Magnetic Resonance Enterography

MRE examinations were performed using a Philips Ingenia 3.0T device (Philips Medical Systems International, Best, Noord-Brabant, The Netherlands). Patients were instructed to fast for at least 6 h before the examination. In addition, they were given 1.5 L of 2.5% mannitol water-based oral solution, which they had to consume 60 min before the actual procedure. No particular preparation of the colon was performed before the examination.

**Figure A3:**
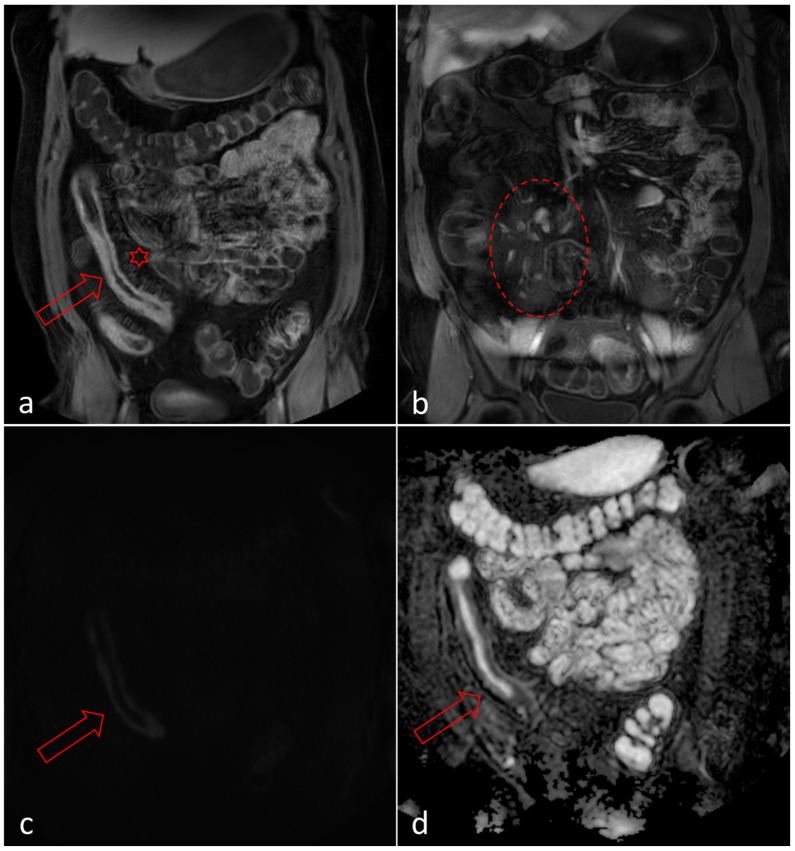
MRE—T1 postcontrast phase (venous phase), coronal plane: (**a**) the arrow indicates an increased wall thickness affecting the terminal ileum continuously, with increased homogeneous contrast uptake and engorged vasa recta in the affected region (star). (**b**) The area inside the circle highlights the presence of multiple enlarged lymph nodes located near the affected terminal ileum. The terminal ileum wall demonstrates restricted diffusion, seen as high signal intensity on the DWI sequence (**c**) and low signal intensity on the ADC map (**d**).

A radiologist with experience in the field of IBD analysed the images obtained at MRE. He was blinded to the results of clinical activity scores, laboratory tests or those obtained from US and CEUS. The assessed parameters included: BWT, the presence of parietal edema, mesenteric fatty proliferation, the existence of bowell wall ulcers, the presence of complications such as stenoses, fistulas or abscesses and the bowel wall enhancement after the administration of gadolinium (Appendix A Figure A3).

Quantification of CD severity by MRE was performed using a score proposed by Ordás et al. [33], called Simplified Magnetic Resonance Index of Activity for Crohn’s Disease (MaRIAs). This score evaluates 4 parameters and gives 1 point each for bowel wall thickness > 3 mm, the presence of bowel wall edema and perienteric fat stranding, and 2 points for the existence of bowel wall ulcers.

A MaRIAs score greater than 1 has been shown to identify intestinal segments with an active CD with 90% sensitivity and 81% specificity, and a score greater than 2 indicates the presence of severe lesions with 85% sensitivity and 92% specificity [33].

### 2.5. Statistical Analysis

The obtained data were analysed using Microsoft Excel (Microsoft Corp., Redmond, WA, USA). The utility of IUS + CEUS and MRE for differentiating active from inactive forms of CD was evaluated by determining the sensitivity, specificity and positive and negative predictive values, using the results of clinical activity scores (CDAI, HBI) and laboratory tests (calprotectin faecal, CRP) as reference methods. Next, we created receiver operating characteristics (ROC) curves using this data and calculated the area under the curve (AUC) for accuracy assessment.

According to the data obtained from the studies of Swets [38], an AUC value between 0.50–0.70 suggests poor accuracy, values between 0.70–0.90 indicate good accuracy, and values above 0.90 offer excellent accuracy.

The Pearson correlation coefficient was used to determine the type of correlation between cross-sectional imaging findings, clinical activity scores, and laboratory tests. To identify the statistical significance of the correlation coefficient obtained after comparing the data, we used the *t*-test (*t*-test). A *p*-value less than 0.05 suggested a statistically significant relationship.

## 3. Results

**Figure A4:**
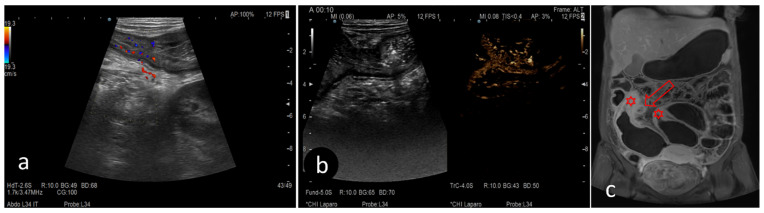
IUS and MRE aspects in a patient with a moderate form of stricturing Crohn’s disease (CDAI = 339; HBI = 9; faecal calprotectin = 2190 μg/g; CRP = 30.51 mg/L). (**a**) IUS: Thickened wall, with typical bowel wall stratification loss, resulting in stenosis with dilation of the intestinal lumen upstream of the obstruction. Colour Doppler imaging highlights the presence of the colour flow signal in both the bowel wall and surrounding mesenteric fat—Limberg score = 4. (**b**) CEUS shows transmural enhancement, suggesting an inflammatory stricture. (**c**) MRE: T1 postcontrast phase (venous phase), coronal plane—the stars highlight stenotic areas with an increased wall thickness affecting the ileum discontinuously, resulting in dilated normal ileum segments between the affected areas. The arrow indicates a possible abscess near one of the affected ileum segments.

During a time interval of 27 months, 44 patients with confirmed ileal CD were included in the study (18 men, 26 women; mean age 43 years, standard deviation (STDEV)—10.34). One female patient was excluded because she could not tolerate the MRE examination due to claustrophobia. The clinical characteristics of the patients are shown in Table 1. From a total of 44 patients, according to the Montreal classification, 8 were known to have a stenotic form of CD. Four patients from this group required surgical intervention for intestinal obstruction before the study. Using the IUS, we could highlight the presence of intestinal stenoses in the case of four patients, the results being later confirmed by the MRE (Appendix A Figure A4). The mean values of the clinical severity scores were 210.54 (STDEV = 84.61) for CDAI, respectively, and 7.04 (STDEV = 3.25) for HBI.

Based on clinical activity scores, CDAI identified 14 patients (31.81%) in clinical remission and 30 patients with active disease (Appendix A Table A1); according to HBI, there were 8 patients (18.18%) with inactive disease and 36 patients with active forms of the disease. The faecal calprotectin and CRP levels revealed 28 (63.63%), respectively, and 25 (56.81%) patients with active disease. Regarding the information obtained at CDI, the Limberg score classified 12 patients (27.27%) as having inactive disease and 32 patients with active disease. At CEUS, the scores proposed by Medellin-Kowalewski et al. [10] and Ripollés et al. [31] identified 4 (9.09%), respectively, 8 (18.18%), patients with inactive disease and 40, respectively, 36 patients with active disease. MaRIAs score, used to quantify the results obtained after MRE, detected 8 patients (18.18%) with inactive disease and 36 patients with active disease, of which 26 patients (59.09%) had severe lesions (Figure 3).

**Figure 4:**
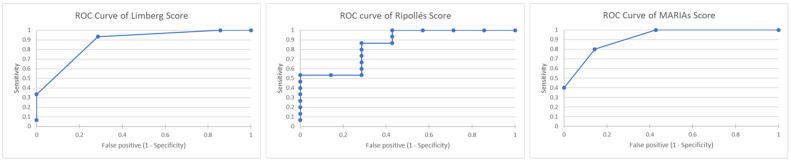
Receiver operating characteristic curves of Limberg score, Ripollés score and MaRIAs score for the diagnosis of active CD using Crohn’s disease activity index as reference method.

Using CDAI as the reference method, the Limberg score presented a sensitivity of 93.33% and a specificity of 71.43% (AUC = 0.98) for differentiating active from inactive forms of the disease (Table 2—a more detailed table version can be consulted in the Appendix A, Table A2 and Table A3). For the score proposed by Ripollés et al. [31] and MaRIAs score, the sensitivity and specificity results were similar, 100.00% and 57.14%, respectively, with an AUC value for Ripollés score of 0.847 and 0.971 for MaRIAs score (Figure 4).

**Figure 5:**
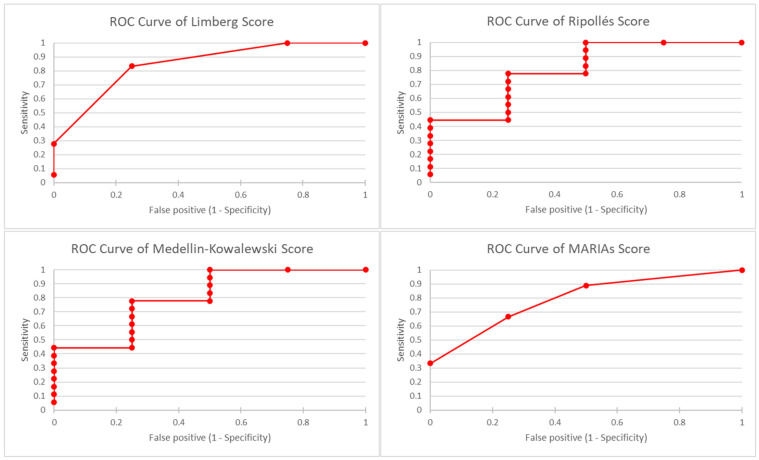
Receiver operating characteristic curves of Limberg score, Ripollés score and MaRIAs score for the diagnosis of active CD using Harvey–Bradshaw Index as reference method.

Compared to HBI (Table 2), Limberg score presented a sensitivity of 83.33% and a specificity of 75.00% (AUC = 0.958) for the evaluation of CD activity and Ripollés and MaRIAs scores had a sensitivity of 88.89% and a specificity of 50.00% (AUC = 0.805 for Ripollés score, respectively, 0.888 for MaRIAs score). In comparison, the score proposed by Medellin-Kowalewski et al. [10] had a sensitivity of 100.00% and a specificity of 50.00% (AUC = 0.805) (Figure 5).

Regarding the use of laboratory tests as reference methods, based on the results of faecal calprotectin (Table 2), the Limberg score was characterised by a sensitivity of 100.00% and a specificity of 83.33% (AUC = 1), and in the case of MaRIAs score, a sensitivity of 100.00% and a specificity of 66.67% (AUC = 1) were achieved for the differentiation of active forms of the disease. The results of the sensitivity and specificity of the imaging tests for assessing CD activity using the CRP levels as the reference method are shown in Table 2 (Appendix A Table A3).

Pearson correlation coefficient values between clinical severity scores, laboratory tests and results of cross-sectional imaging techniques are shown in Table 3 (a more detailed table version can be consulted in the Appendix A, Table A4 and Table A5). Our study demonstrated a good correlation between CDAI and Limberg score (r = 0.667, 95% confidence interval (CI) [0.46, 0.8], *p* < 0.001) and between CDAI and MaRIAs score (r = 0.614, 95% CI [0.39, 0.77], *p* < 0.001) and a reasonable correlation between CDAI and Medellin-Kowalewski score (r = 0.537, 95% CI [0.29, 0.72], *p* < 0.001). Between HBI and imaging investigations, the results demonstrated reasonable correlations for most of the evaluated scores, the highest Pearson correlation coefficient value being obtained for HBI and MaRIAs score (r = 0.594, 95% CI [0.36, 0.76], *p* < 0.001). In addition, the study identified a good correlation between faecal calprotectin values and MaRIAs score (r = 0.697, 95% CI [0.46, 0.84], *p* < 0.001). In the case of CRP, only weak correlations with the scores assessed were demonstrated.

## 4. Discussion

A consensus has yet to be established regarding the treatment goals in CD. Both achieving clinical remission and mucosal healing are discussed. There is often a discrepancy between the symptoms reported by CD patients and the results of laboratory tests or imaging investigations. Colonoscopy is currently an indispensable method for monitoring patients with CD. One of the most used endoscopic scores in clinical practice is represented by the Simple Endoscopic Score for Crohn’s Disease (SES-CD) (a score between 0–2 suggests inactive disease) [39]. Results are conflicting regarding the correlation of SES-CD with various clinical severity scores, such as the CDAI, with some studies suggesting a weak correlation between the two methods [40,41]. The levels of some inflammatory biomarkers, such as CRP and faecal calprotectin, seem to show good correlations with the endoscopic activity of colonic or ileocolonic CD, but not in patients with lesions limited to the ileum [19,42,43,44]. Therefore, such biomarkers should be used as initial methods to identify patients who require more complex investigations but often find utility in monitoring disease activity and response to drug treatment [5]. A meta-analysis that included 13 studies (1471 patients with IBD) demonstrated that a faecal calprotectin threshold value of 250 μg/g presents a specificity of 82% for identifying active forms of IBD, which is superior to values corresponding to a threshold of 100 μg/g or 50 μg/g (specificity of 66% and 60%, respectively) [45]. However, faecal calprotectin appears to be associated with a more remarkable ability to assess disease activity in ulcerative colitis than CD [45].

The ileum cannot always be explored endoscopically. A study evaluating 189 patients with CD using ileocolonoscopy and CTE found that 53.7% of patients with normal endoscopic aspects had an active CD of the small bowel [46]. For this reason, all newly diagnosed CD patients should benefit from small bowel evaluation by complementary radiological methods, such as MRE, CTE or IUS, to assess the disease’s extent, activity and the presence of complications [47].

Cross-sectional imaging techniques provide information on the bowel wall and surrounding tissues, thus supplying a superior disease phenotype classification [4]. Studies have demonstrated similar sensitivity and specificity for CT and MRI to diagnose small bowel lesions [14,48,49]. Since most CD patients are diagnosed at young ages due to the lack of ionising radiation, MRI is preferable if this investigation is available [4,47]. The MRI examination of the pelvis is instrumental in describing the anatomy of perianal fistulas as an auxiliary method to the examination under anaesthesia (EUA) of the anorectal region by an experienced surgeon, currently representing the method of choice for the evaluation of perianal CD [4]. In addition to the diagnostic role, MRI can also be used to assess CD activity. In this regard, several scores have been developed, some of which have been validated in clinical practice [50,51,52,53]. Among the most used is the Magnetic Resonance Index of Activity (MaRIA), which shows a good correlation with the Crohn’s Disease Endoscopic Index of Severity (CDEIS) [51,54].

In recent years there has been a growing interest in using IUS and CEUS as alternatives to MRI for evaluating CD patients. IUS is a non-irradiating method, being particularly useful in the case of young patients. In addition, it is cheap and widely available, readily accepted by patients, and thus can be repeated as often as needed. It has the disadvantage of being operator-dependent, and experience in evaluating the digestive tract is limited [55]. A study evaluating the learning curve of IUS for the evaluation of patients with IBD concluded that at least 150 supervised examinations are required to acquire proficiency in IUS, with physicians already experienced in abdominal ultrasonography developing these skills more quickly than those without experience [56]. Most patients prefer IUS to MRE. MRE examination is associated with more significant discomfort and longer post-procedural recovery than IUS, especially in young or emotional patients [57]. However, the discomfort associated with the procedure seems to matter less than the diagnostic accuracy of the investigation [57]. Several meta-analyses have demonstrated similar results between IUS, CTE or MRE, with sensitivity and specificity between 85–95% regarding the diagnosis of CD [11,12,13,14,15]. IUS seems inferior to MRI for establishing the extension of the disease in the small bowel and characterising proximal lesions or lesions localised in the pelvis [7,14]. Still, the diagnostic accuracy of complications such as stenoses, fistulas or abscesses is similar between the methods [7,14]. In our study, most patients had lesions limited to the terminal ileum, which is why no great difficulties were identified in evaluating the segments affected by the inflammatory process by ultrasonography. CD’s most frequent and constant ultrasonographic feature is the thickening of the intestinal wall above the threshold value of 3–4 mm, directly proportional to the disease severity [58]. A meta-analysis by Fraquelli et al. [59] demonstrated that, although the BWT threshold of 4 mm has a lower sensitivity than the value of 3 mm, it is associated with a higher specificity for Crohn’s disease. Numerous studies have demonstrated a good correlation between BWT assessed by ultrasonography and disease activity assessed by colonoscopy [37,59,60]. Increased BWT and loss of parietal layering, identified via IUS, appear to be associated with a higher risk of surgery in CD patients [37,61,62].

Recently, there has been increasing talk of transmural healing (TH) as a therapeutic goal in CD [63]. Achieving TH appears to be associated with a more favourable long-term outcome than mucosal healing [64]. For this reason, some authors propose that TH represents the main therapeutic target in CD [63]. At the moment, there is yet to be a universally valid definition of this term. TH can be assessed through MRE, CTE or IUS. Parameters used to define TH could include BWT and parietal vascularisation assessed by CDI or bowel wall enhancement of contrast agents [63]. However, using BWT as the sole criterion for defining TH seems insufficient, as this could result from fibrosis, which is why additional evaluation of the vascular pattern is recommended [63,65]. CDI can evaluate the inflammatory activity of the bowel wall by assessing the vascular signal provided by the larger blood vessels [23,25,66]. According to the classic Limberg classification, the degree of the colour Doppler signal is usually measured at the level of the thickened bowel wall (stage ≥ 2), but modified versions of this score have been proposed that evaluate the presence of the signal also at the level of the normal looking bowel wall, considering punctiform Doppler signal as non-pathological or uncertain [67,68,69,70]. In our study, the colour Doppler signal was identified only in association with bowel wall thickening of >4 mm, consistent with the classic Limberg score. Most likely, these modified variants of the classification were proposed to be adapted to modern ultrasonographic systems that associate a better image resolution. Using contrast agents during CEUS allows a superior evaluation of the inflammation by assessing the bowel wall vascularisation at the microcirculatory level, the neoangiogenesis of the bowel wall is characteristic of the active inflammation associated with CD [10,29,71,72].

Numerous studies, including those that provided the scores used in our research, have demonstrated a good correlation between disease severity assessed by cross-sectional imaging techniques and endoscopic CD activity [10,26,29,31,32,33,34,73,74]. Ripollés et al. [31] confirm that the primary ultrasonographic criterion for evaluating CD activity is represented by the bowel wall enhancement after intravenous administration of contrast agents, describing a sensitivity of 92% and a specificity of 86.4% for this parameter. In addition, TH also seems to show a good correlation with faecal calprotectin and CRP levels [63]. Cerrillo et al. [75] described a sensitivity of 90% and a specificity of 74%, corresponding to a faecal calprotectin cut-off level of 166.50 μg/g for the diagnosis of inflammation in ileal CD, using MRE as the reference method. Regarding the correlation with clinical activity scores, the results available in the literature are contradictory. Recently, Yamanashi et al. [76] described a strong correlation between an ultrasonographic score (based on BWT, parietal stratification, presence of stenoses, colour Doppler signal and mesenteric fat appearance) proposed for assessing the severity of CD and CDAI, even superior to the correlation established for SES-CD and CDAI. Also, Yiğit et al. [74] identified a good correlation between Limberg score, bowel wall thickness and mesenteric inflammation assessed through IUS or CTE and clinical activity scores (CDAI and HBI). However, other studies have suggested that quantitative measurement of bowel wall vascularisation utilising CEUS or MRI shows a weak or no correlation with clinical activity scores or laboratory tests, such as CRP levels [77,78].

Our study aimed to identify the correlation between various scores proposed in the literature for assessing CD activity by IUS + CEUS or MRI and clinical activity scores (CDAI, HBI) or specific laboratory tests (faecal calprotectin, CRP). The scores were selected according to the degree of difficulty of their calculation, the simplicity and the quickness of obtaining a result being essential elements in everyday clinical practice. Time optimisation could be achieved by integrating dedicated quantitative analysis software, such as the one used in our study, in ultrasonography systems, without the need for image recording and subsequent processing on other devices. Compared to CDAI, the best correlation was identified for the Limberg score, followed by MaRIAs score, the simple US score and then the score proposed by Medellin-Kowalewski and colleagues. A sensitivity of 93.33% and a specificity of 71.43% (AUC = 0.98) were demonstrated in the case of the Limberg score for differentiating patients with active forms of CD from those in remission and for MaRIAs score a sensitivity of 100.00% and a specificity of 57.14% (AUC = 0.97). BWT showed a reasonable correlation with CDAI but was still inferior to the Limberg score, reinforcing the idea that the additional assessment of the vascular pattern provides superior information for the evaluation of disease activity. Regarding HBI, the best correlation was observed for MaRIAs score, followed by the Limberg score, the Medellin-Kowalewski score, and the simple US score. Faecal calprotectin showed the best correlation also with MaRIAs score. Still, in the case of CRP, there was only a weak correlation for all evaluated scores, which shows that more than this investigation is needed to assess the disease activity.

Other authors also confirmed similar results for MaRIAs score. Roseira et al. [79] demonstrated a strong correlation between this score and faecal calprotectin (R = 0.88, *p* < 0.001) in patients with ileal CD. Regarding CEUS, in our study, the score proposed by Medellin-Kowalewski et al. which uses the PE parameter showed reasonable correlations with clinical activity scores but only a weak correlation with inflammatory biomarkers. Also, Freitas et al. [80] reported a good correlation between PE and HBI, with PE being significantly different in patients with clinically active disease (HBI ≥ 5) compared to those with inactive forms. In contrast, no statistically significant correlations were identified with CRP or faecal calprotectin levels. For that reason, although CEUS appears to be a promising method, there are currently insufficient data to recommend its use as a first-line investigation.

Taking an overview, we can see that the assessment of CD activity by MRI using MaRIAs score shows overall the best correlations with clinical severity scores and inflammatory biomarkers, especially CDAI and faecal calprotectin. Favourable results were also obtained for the ultrasonographic scores, particularly the Limberg score, which is even superior to MaRIAs score in terms of specificity for diagnosing active forms of CD, according to the reference methods used. Therefore, following international guidelines, we suggest using MRI for the initial evaluation of patients and, subsequently, IUS for their follow-up.

The main limitation of our study concerns the small number of patients evaluated, so the results obtained may not be reliable in the case of larger groups. Thus, additional multicenter studies that include more subjects are needed. At the same time, another limitation could be that we used only one type of contrast agent during CEUS, especially since in the study conducted by Medellin-Kowalewski et al., another contrast agent was used. Thus, there could be a discrepancy between the PE values obtained in our research and the cut-off value proposed by the authors of the score. It should also be emphasised that this threshold could be a device-dependent parameter, possibly obtaining different results when using different settings and ultrasound systems. Ileocolonoscopy remains the gold standard for diagnosing and monitoring patients with CD. Therefore, another limitation of our study is that we could not compare the results of the assessed imaging investigations with the endoscopic activity scores in the case of all patients included. It should be noted that only 26 of 44 patients agreed to be evaluated by ileocolonoscopy. Referring to this number of patients, the best correlation with SES-CD was identified for MaRIAs score (r = 0.562, 95% CI [0.22, 0.78], *p* < 0.01), then for the Limberg score (r = 0.382, 95% CI [−0.0056, 0.67], *p* > 0.05), but only a very weak correlation was observed for the other evaluated scores. Regarding the accuracy of diagnosing active forms of the disease using SES-CD as a reference method, for MaRIAs score, a sensitivity of 100.00% and a specificity of 66.67% (AUC = 0.933) were established. In comparison, the Limberg score had a sensitivity of 90.00% and a specificity of 66.67% (AUC = 0.667).

Considering that more and more authors propose transmural healing as the primary goal of Crohn’s disease therapy, we can conclude that cross-sectional imaging techniques represent viable methods for evaluating disease activity. Thus, the prognosis of patients with Crohn’s disease could be improved by monitoring with the help of non-invasive imaging investigations, widely available and easily tolerated by patients, such as ultrasonography. Although magnetic resonance does not appear to be superior to ultrasonography in terms of accuracy for differentiating active forms of Crohn’s disease from those in remission, the results of our study suggest that magnetic resonance associates a better correlation with clinical severity scores and faecal calprotectin levels compared to ultrasonography. However, more studies are needed to validate these results.

## Figures and Tables

**Table 1:** Clinical characteristics of the patients (n = 44).

Age [mean (range)]	43 (23–66)
Gender (male/female)	18/26
**Disease behaviour**	
Non-stricturing/non-penetrating	36
Stricturing	8
Penetrating	0
- Perianal disease	2
**Crohn’s Disease Activity Index** [mean (STDEV)]	210.54 (84.61)
**Harvey–Bradshaw Index** [mean (STDEV)]	7.04 (3.25)

STDEV—standard deviation.

**Table 2:** Assessment of the quality of IUS + CEUS and MRE in detecting active forms of Crohn’s disease using Crohn’s disease activity index, Harvey–Bradshaw Index, fecal calprotectin test and C-reactive protein test as reference methods.

	US—Maximal Wall Thickness			Limberg Score			Ripollés Score			MaRIAs Score		
	Sen (%)	Spe (%)	AUC	Sen (%)	Spe (%)	AUC	Sen (%)	Spe (%)	AUC	Sen (%)	Spe (%)	AUC
Crohn’s disease activity index	100	14.29	0.87	93.33	71.43	0.98	100	57.14	0.84	100	57.14	0.97
Harvey–Bradshaw Index	100	25	0.88	83.33	75%	0.95	88.89	50	0.80	88.89	50	0.88
Faecal calprotectin	100	16.67	0.9	100	83.33	1	100	66.67	0.86	100	66.67	1
C-reactive protein	100	12.50	0.72	90.91	50	0.88	100	37.50	0.71	100	37.50	0.88

**Table 3:** Correlation values and significance of results in comparison between clinical activity scores (Crohn’s disease activity index/Harvey–Bradshaw Index), laboratory tests (fecal calprotectin/C-reactive protein), IUS + CEUS and MRI.

	US—Maximal Wall Thickness		Limberg Score		Medellin-Kowalewski Score		MaRIAs Score	
	r	*p*-Value	r	*p*-Value	r	*p*-Value	r	*p*-Value
Crohn’s disease activity index	0.502	<0.001	0.667	<0.001	0.537	<0.001	0.614	<0.001
Harvey–Bradshaw Index	0.352	<0.05	0.468	<0.01	0.459	<0.01	0.594	<0.001
Faecal calprotectin	0.419	<0.05	0.446	<0.05	0.159	>0.05	0.697	<0.001
C-reactive protein	0.195	>0.05	0.238	>0.05	0.298	>0.05	0.395	<0.05

r—Pearson correlation coefficient.

## Data Availability

The data used to support the findings of this study are available from the corresponding author upon reasonable request.

## Appendix A

**Table A1:** Disease activity based on results of clinical activity scores, laboratory tests and imaging techniques.

Parameters Evaluated	Disease’s Activity—No. of Patients (%)			
	Inactive	Active		
Maximal wall thickness (mm)	<4 mm: 2 (4.54%)	Mild (4.0–6.0): 10 (22.73%)	Moderate (6.1–8.0): 26 (59.09%)	Severe (≥8.1): 6 (13.63%)
Limberg Score	Grade 0 and Grade 1: 12 (27.27%)	Grade 2: 22 (50%)	Grade 3: 8 (18.18%)	Grade 4: 2 (4.54%)
Ripollés Score	Inactive disease < 8.38: 8 (18.18%)	Active disease > 8.38: 36 (81.81%)		
Simple CEUS Score	Inactive disease < 8.38: 6 (13.63%)	Active disease > 8.38: 38 (86.36%)		
Simple US score	Inactive disease < 5.5: 2 (4.54%)	Active disease > 5.5: 42 (95.45%)		
Medellin-Kowalewski Score	Inactive < 18.2 dB: 4 (9.09%)	Mild to moderate 18.2–22.8 dB: 8 (18.18%)	Moderate to severe >22.8 dB: 32 (72.72%)	
MaRIAs	Inactive < 1: 8 (18.18%)	Active disease ≥ 1: 10 (22.72%)	Severe lesions ≥ 2: 26 (59.09%)	
Crohn’s disease activity index	Remission (<150): 14 (31.81%)	Mild (150–220): 10 (22.72%)	Moderate (220–450): 20 (45.45%)	Severe (>450): 0 (0%)
Harvey–Bradshaw Index	Remission (<5): 8 (18.18%)	Mild (5–7): 16 (36.36%)	Moderate (8–16): 20 (45.45%)	Severe (>16): 0 (0%)
Faecal calprotectin (μg/g)	Inactive (<250 μg/g): 16 (36.36%)	Active (> 250 μg/g): 28 (63.63%)		
CRP (mg/L)	Inactive (<10 mg/L): 19 (43.18%)	Active (>10 mg/L): 25 (56.81%)		

**Table A2:** Assessment of the quality of IUS + CEUS and MRE in detecting active forms of Crohn’s disease using Crohn’s disease activity index and Harvey–Bradshaw Index as reference methods.

Parameters	Sensitivity (%)	Specificity (%)	AUC	Positive Predictive Value (%)	Negative Predictive Value (%)
US—maximal wall thickness vs. CDAI	100%	14.29%	0.876	71.43%	100%
Limberg Score vs. CDAI	93.33%	71.43%	0.980	87.50%	83.33%
Ripollés Score vs. CDAI	100%	57.14%	0.847	83.33%	100%
Simple CEUS Score vs. CDAI	100%	42.86%	0.847	78.95%	100%
Simple US Score vs. CDAI	100%	14.29%	0.895	71.43%	100%
Medellin-Kowalewski Score vs. CDAI	100%	28.57%	0.828	75%	100%
MaRIAs Score vs. CDAI	100%	57.14%	0.971	83.33%	100%
US—maximal wall thickness vs. HBI	100%	25%	0.888	85.71%	100%
Limberg Score vs. HBI	83.33%	75%	0.958	93.75%	50%
Ripollés Score vs. HBI	88.89%	50%	0.805	88.89%	50%
Simple CEUS Score vs. HBI	94.44%	50%	0.805	89.47%	66.67%
Simple US Score vs. HBI	100%	25%	0.916	85.71%	100%
Medellin-Kowalewski Score vs. HBI	100%	50%	0.805	90%	100%
MaRIAs Score vs. HBI	88.89%	50%	0.888	88.89%	50%

**Table A3:** Assessment of the quality of IUS + CEUS and MRE in detecting active forms of Crohn’s disease using faecal calprotectin test and C-reactive protein test as reference methods.

Parameters	Sensitivity (%)	Specificity (%)	AUC	Positive Predictive Value (%)	Negative Predictive Value (%)
US—maximal wall thickness vs. FC	100%	16.67%	0.9	66.67%	100%
Limberg Score vs. FC	100%	83.33%	**1**	90.91%	100%
Ripollés Score vs. FC	100%	66.67%	0.866	83.33%	100%
Simple CEUS Score vs. FC	100%	50%	0.866	76.92%	100%
Simple US Score vs. FC	100%	16.67%	0.9	66.67%	100%
Medellin-Kowalewski Score vs. FC	100%	33.33%	0.833	71.43%	100%
MaRIAs Score vs. FC	100%	66.67%	**1**	83.33%	100%
US—maximal wall thickness vs. CRP	100%	12.50%	0.727	61.11%	100%
Limberg Score vs. CRP	90.91%	50%	0.886	71.43%	80%
Ripollés Score vs. CRP	100%	37.50%	0.715	68.75%	100%
Simple CEUS Score vs. CRP	100%	25%	0.715	25%	100%
Simple US Score vs. CRP	100%	12.50%	0.750	61.11%	100%
Medellin-Kowalewski Score vs. CRP	100%	12.50%	0.715	61.11%	100%
MaRIAs Score vs. CRP	100%	37.50%	0.886	68.75%	100%

FC—faecal calprotectin; CRP—C-reactive protein.

**Table A4:** Correlation values and significance of results in comparison between clinical activity scores (Crohn’s disease activity index/Harvey–Bradshaw Index), IUS + CEUS and MRI.

Parameters	Pearson Correlation Coefficient	95% Confidence Interval	*p-*Value
CDAI vs. US—maximal wall thickness	0.502	[0.24, 0.7]	<0.001
CDAI vs. Limberg score	0.667	[0.46, 0.8]	<0.001
CDAI vs. Ripollés score	0.320	[0.026, 0.56]	<0.05
CDAI vs. Simple CEUS score	0.324	[0.031, 0.57]	<0.05
CDAI vs. Simple US score	0.598	[0.37, 0.76]	<0.001
CDAI vs. Medellin-Kowalewski score	0.537	[0.29, 0.72]	<0.001
CDAI vs. MaRIAs	0.614	[0.39, 0.77]	<0.001
HBI vs. US—maximal wall thickness	0.352	[0.062, 0.59]	<0.05
HBI vs. Limberg score	0.468	[0.2, 0.67]	<0.01
HBI vs. Ripollés score	0.184	[−0.12, 0.46]	>0.05
HBI vs. Simple CEUS score	0.186	[−0.12, 0.46]	>0.05
HBI vs. Simple US score	0.420	[0.14, 0.64]	<0.01
HBI vs. Medellin-Kowalewski score	0.459	[0.19, 0.67]	<0.01
HBI vs. MaRIAs	0.594	[0.36, 0.76]	<0.001

**Table A5:** Correlation values and significance of results in comparison between laboratory tests (faecal calprotectin/C-reactive protein), IUS + CEUS and MRI.

Parameters	Pearson Correlation Coefficient	95% Confidence Interval	*p-*Value
FC vs. US—maximal wall thickness	0.419	[0.083, 0.67]	<0.05
FC vs. Limberg score	0.446	[0.12, 0.69]	<0.05
FC vs. Ripollés score	0.076	[−0.28, 0.41]	>0.05
FC vs. Simple CEUS score	0.079	[−0.28, 0.42]	>0.05
FC vs. Simple US score	0.395	[0.054, 0.65]	<0.05
FC vs. Medellin-Kowalewski score	0.159	[−0.2, 0.48]	>0.05
FC vs. MaRIAs	0.697	[0.46, 0.84]	<0.001
CRP vs. US—maximal wall thickness	0.195	[−0.13, 0.48]	>0.05
CRP vs. Limberg score	0.238	[−0.088, 0.52]	>0.05
CRP vs. Ripollés score	0.030	[−0.29, 0.35]	>0.05
CRP vs. Simple CEUS score	0.031	[−0.29, 0.35]	>0.05
CRP vs. Simple US score	0.213	[−0.11, 0.5]	>0.05
CRP vs. Medellin-Kowalewski score	0.298	[−0.023, 0.56]	>0.05
CRP vs. MaRIAs	0.395	[0.087, 0.64]	<0.05

FC—faecal calprotectin; CRP—C-reactive protein.
